# A Simple Feeding Efficiency Test as a Screening Tool for Oral Function in Older Adults: A Cross-Sectional Study

**DOI:** 10.7759/cureus.101046

**Published:** 2026-01-07

**Authors:** Kano Kawamura, Norio Kawamura, Kanako Kawamura, Inho Soh, Madoka Funahara

**Affiliations:** 1 Oral Health Sciences, Graduate School of Dentistry, Kyushu Dental University, Kitakyushu, JPN; 2 Dentistry, Kawamura Dental Clinic, Shimanto, JPN; 3 Oral Health Sciences, Kawamura Dental Clinic, Shimanto, JPN; 4 Oral Health Sciences, Kyushu Dental University, Kitakyushu, JPN

**Keywords:** feeding efficiency, older adults, oral frailty, oral function, salivary bacteria

## Abstract

Background

Aspiration pneumonia is a major public health concern among older adults in aging societies. Although various oral function tests are available, most assess only a single aspect of oral function and require specialized equipment. Therefore, a simple and comprehensive screening indicator applicable in routine clinical practice is needed. This study aimed to evaluate the potential usefulness of a newly developed feeding efficiency (FE) test as a screening indicator of oral function in older adults.

Methods

This cross-sectional study included older adults aged ≥65 years who visited a dental clinic and provided informed consent. Data collected included age, sex, oral hygiene status (Oral Hygiene Index-Debris Index (OHI-DI)), salivary flow rate, maximum tongue pressure, oral diadochokinesis (ODK) (/pa/, /ta/, /ka/), number of remaining teeth, and functional tooth units of natural teeth and artificial teeth on implant-supported or fixed pontics (nif-FTU). FE was assessed using a sausage-based feeding task and was defined as the amount of food ingested (g) divided by the number of chewing cycles required to complete swallowing, reflecting FE during habitual eating. Associations between FE, oral function measures, and salivary bacterial counts were analyzed.

Results

A total of 43 participants (16 males and 27 females; mean age 73.7 years) were included. FE values ranged from 5.6 to 67.7 (mean±SD: 24.4±14.8). Participants with nif-FTU ≥6 demonstrated significantly higher FE values than those with nif-FTU <6 (*p*=0.023). No significant associations were observed between salivary bacterial counts and tongue pressure, ODK, or nif-FTU. In contrast, salivary bacterial counts were significantly higher in participants with FE <15 (*p*=0.045).

Conclusions

The FE test may capture aspects of overall oral function during the feeding process that are not fully reflected by conventional oral function measures. Low FE values were associated with increased salivary bacterial counts, suggesting potential relevance to aspiration pneumonia risk. The FE test may have potential as a simple and practical screening indicator for oral functional decline in older adults; however, further studies involving frailer populations and longitudinal designs are warranted to validate its clinical applicability.

## Introduction

Japan is one of the most rapidly aging societies in the world, and aspiration pneumonia among older adults has become a major public health concern [1].

Prevention of aspiration pneumonia is critically important for extending healthy life expectancy. Aspiration pneumonia is considered to develop through the aspiration of saliva containing pathogenic microorganisms, combined with age-related or underlying disease-related declines in immune function [2,3]. Accordingly, the importance of oral hygiene management has been widely recognized, and previous studies have demonstrated that appropriate oral care can reduce the incidence of aspiration pneumonia [4,5]. However, a high risk of aspiration pneumonia has also been reported in edentulous older adults with minimal dental plaque accumulation [6]. In addition, our previous studies demonstrated that tongue function declines with a reduction in the number of functional teeth [7] and that among older adults requiring long-term care, reduced tongue pressure is associated with increased salivary bacterial counts, potentially contributing to the development of aspiration pneumonia [8]. These findings suggest that, in addition to oral hygiene management, the maintenance of oral function plays a crucial role in aspiration pneumonia prevention.

Oral function represents the coordinated capacity required for eating-related activities, including masticatory force, tongue movement, and swallowing. In clinical practice, oral function is commonly evaluated using individual assessments such as tongue pressure measurement, occlusal force measurement, masticatory performance tests (e.g., the gummy jelly method), and swallowing function tests, including videoendoscopic evaluation of swallowing (VE). However, these assessments evaluate isolated components of oral function and do not easily capture the entire functional process from food intake to swallowing. Moreover, many of these examinations require specialized equipment or technical expertise and are often conducted only after oral functional decline has become apparent. Because early detection and intervention are essential for preventing oral frailty, there is a strong need for simple screening indicators that can be readily implemented in routine clinical practice and community settings. In the present study, these conventional oral function tests were used to assess specific components of oral function and to examine how each component relates to feeding efficiency (FE) as a comprehensive indicator of oral function during eating.

Based on the hypothesis that individuals with preserved oral function can chew food efficiently and swallow it smoothly, we developed an FE test using a wiener sausage, defined as the amount of food consumed divided by the number of chewing cycles. This test was designed as a conceptual screening indicator reflecting the overall efficiency of habitual feeding behavior rather than a detailed physiological assessment of individual oral functions. Because impairments in any component of mastication, tongue movement, or swallowing may reduce FE, FE may provide a comprehensive reflection of oral function during eating. Therefore, the aim of this study was to investigate the feasibility of FE as a screening indicator of oral function by examining its associations with tongue pressure, functional tooth units (FTUs), and salivary bacterial counts, which are clinically relevant to aspiration pneumonia risk.

Based on an exploratory framework, we prespecified the hypothesis that FE would reflect overall oral function during eating and be associated with salivary bacterial counts.

## Materials and methods

Participants

This cross-sectional study included adults aged 60 years or older who visited a dental clinic in Japan between July and December 2024. All participants received a full explanation of the study objectives and provided written informed consent prior to enrollment.

Eligible participants were recruited consecutively during the study period. Exclusion criteria included the inability to expectorate saliva for sample collection and cognitive impairment that prevented reliable measurement of tongue pressure.

Participants were recruited from a private general dental clinic that provides outpatient dental care to community-dwelling older adults. The clinic primarily offers routine dental treatment, preventive care, and prosthodontic services and does not specialize in dysphagia rehabilitation.

Data collection

For all participants, the following variables were assessed: age, sex, oral hygiene status evaluated using the OHI-DI [9], salivary flow rate, maximum tongue pressure, oral diadochokinesis (ODK), the number of remaining teeth, and functional tooth units of natural teeth and artificial teeth on implant-supported or fixed pontics (nif-FTU).

Salivary flow rate was measured using the Saxon test [10]. Before measurement, participants were instructed to keep the oral cavity at rest. A piece of sterile gauze was placed in the mouth, and participants were asked to chew it for 2 min. The gauze was weighed before and after chewing using an electronic balance, and the difference in weight was calculated as the salivary secretion volume (g/2 min).

Tongue pressure was measured using a tongue pressure measurement device (TPM-02E; JMS Co., Ltd., Hiroshima, Japan) [11]. Participants were instructed to hold the balloon probe between the tongue and the palate and compress it with maximal effort. Measurements were performed three times, and the maximum value was adopted as the representative value.

ODK was assessed using the Kenkokun Handy II (TKK3352; SANKA Co., Ltd., Niigata, Japan). Participants were instructed to repeat the syllables /pa/, /ta/, and /ka/ as quickly and clearly as possible for 5 sec each. The number of syllable repetitions per second (times/s), automatically analyzed by the device, was recorded as the representative value [12].

The number of FTUs was defined based on the number of opposing premolars or molars. Specifically, one pair of opposing premolars was defined as 1 FTU, and one pair of opposing molars was defined as 2 FTUs. Accordingly, when all premolars and molars were in occlusion, the total number of FTUs was 12. Nif-FTU included FTUs consisting of natural teeth and implant-supported or fixed prostheses [13].

Feeding efficiency measurement

FE was assessed using a feeding task with a wiener sausage measuring 23 mm in diameter and 15 cm in length (Maruha Nichiro Corporation, Tokyo, Japan). Participants were instructed to chew the sausage freely, reflecting their habitual eating behavior, and the number of chewing cycles required until swallowing was completed was recorded. Swallowing completion was defined as the participant’s subjective confirmation of having swallowed the bolus. The amount of food consumed was calculated by subtracting the weight of the remaining sausage after swallowing from the weight measured before the task. FE was defined as the weight of the consumed sausage (g) divided by the number of chewing cycles required to complete swallowing.

Measurement of salivary bacterial counts

Participants were instructed to expectorate 0.1 mL of saliva, from which deoxyribonucleic acid (DNA) was extracted. Total bacterial load was quantified by real-time polymerase chain reaction (PCR) using universal primers targeting the 16S ribosomal RNA gene. The total bacterial count was calculated based on a standard curve generated using synthetic DNA. The primers, synthetic DNA, and PCR reaction conditions were prepared and performed as previously described [14,15].

Data recording and management

All measurements, including FE, tongue pressure, ODK, FTUs, salivary flow rate, and salivary bacterial counts, were recorded at the time of assessment using standardized case report forms. The collected data were subsequently entered into a secure digital database on password-protected computers. To ensure data quality, data entry was independently checked by two investigators, and discrepancies were resolved by reviewing the original source data. Salivary bacterial count data obtained by real-time PCR were exported directly from the analysis software and verified against sample identification numbers. All participant data were anonymized prior to analysis, and each participant was assigned a unique study identification code. The correspondence table linking personal identifiers and study IDs was stored separately in a secure location. Data access was restricted to authorized members of the research team only, in accordance with institutional data protection policies.

Statistical analysis

All statistical analyses were performed using IBM SPSS Statistics version 26.0 (IBM Japan, Ltd., Tokyo, Japan). Tongue pressure was dichotomized into ≥20 kPa and <20 kPa, based on a previous report indicating an increased risk of aspiration pneumonia in older adults below this threshold [16]. ODK results were expressed as the sum of the repetitions of /ta/ and /ka/ (ta+ka) and categorized into two groups (≥6.0 and <6.0) according to the position paper of the *Japanese Society of Gerodontology* (2016) [17]. The nif-FTU score was categorized into ≥6 and <6.

Because FE is a newly developed indicator with no established reference values, the FE cut-off value was determined based on the distribution of the observed data in this exploratory study. The FE score was therefore categorized into ≥15 and <15 to facilitate exploratory group comparisons rather than to define a clinically validated threshold.

Differences in FE according to tongue pressure, ta+ka, and nif-FTU, as well as differences in salivary bacterial counts according to tongue pressure, ta+ka, nif-FTU, and FE, were analyzed using the Mann-Whitney U test.

Because this was an exploratory study with a limited sample size, subgroup analyses and sensitivity analyses were not planned or performed. Measures of precision, such as confidence intervals, were not calculated, and statistical significance was assessed using p-values derived from non-parametric tests.

Potential confounding factors considered in this study included age, sex, oral hygiene status, salivary flow rate, and the number of FTUs. However, no multivariable adjustment was performed because this study was exploratory in nature and had a limited sample size.

All assessment tools and indices used in this study, including the Oral Hygiene Index-Debris Index (OHI-DI), the Saxon test, ODK, tongue pressure measurement, and FTUs, are widely used clinical or research tools and do not require special licensing for academic use.

Sample size consideration

This study was conducted as an exploratory, cross-sectional study to assess the feasibility and potential clinical relevance of a newly developed FE test. Therefore, a formal a priori sample size calculation was not performed. The sample size was determined based on feasibility, including the number of eligible older adults who attended the dental clinic during the study period and agreed to participate.

Selection/measurement bias

Efforts were made to minimize potential sources of bias. Selection bias was reduced by recruiting consecutive ambulatory older adults who visited the dental clinic during the study period and met the inclusion criteria. Measurement bias was minimized by using standardized and widely accepted assessment tools, including tongue pressure measurement, ODK, and FTUs, all of which were performed according to predefined protocols. All assessments were conducted by trained examiners. However, as this was a cross-sectional exploratory study conducted at a single clinic, residual confounding and selection bias could not be fully eliminated.

Ethics

This study was conducted in accordance with the ethical principles of the Declaration of Helsinki. Ethical approval was obtained from the Institutional Review Board of Kyushu Dental University (approval number: 25-11). Written informed consent for participation in the study and publication of the results was obtained from all participants. The study was registered with the University Hospital Medical Information Network Clinical Trials Registry (UMIN-CTR) (registration number: UMIN000054849; September 24, 2024).

Funding

This study was supported by research funds allocated to graduate students of Kyushu Dental University. The funding source had no role in the study design, data collection, analysis, interpretation of data, or manuscript preparation.

## Results

Participant characteristics

**Figure 1:**
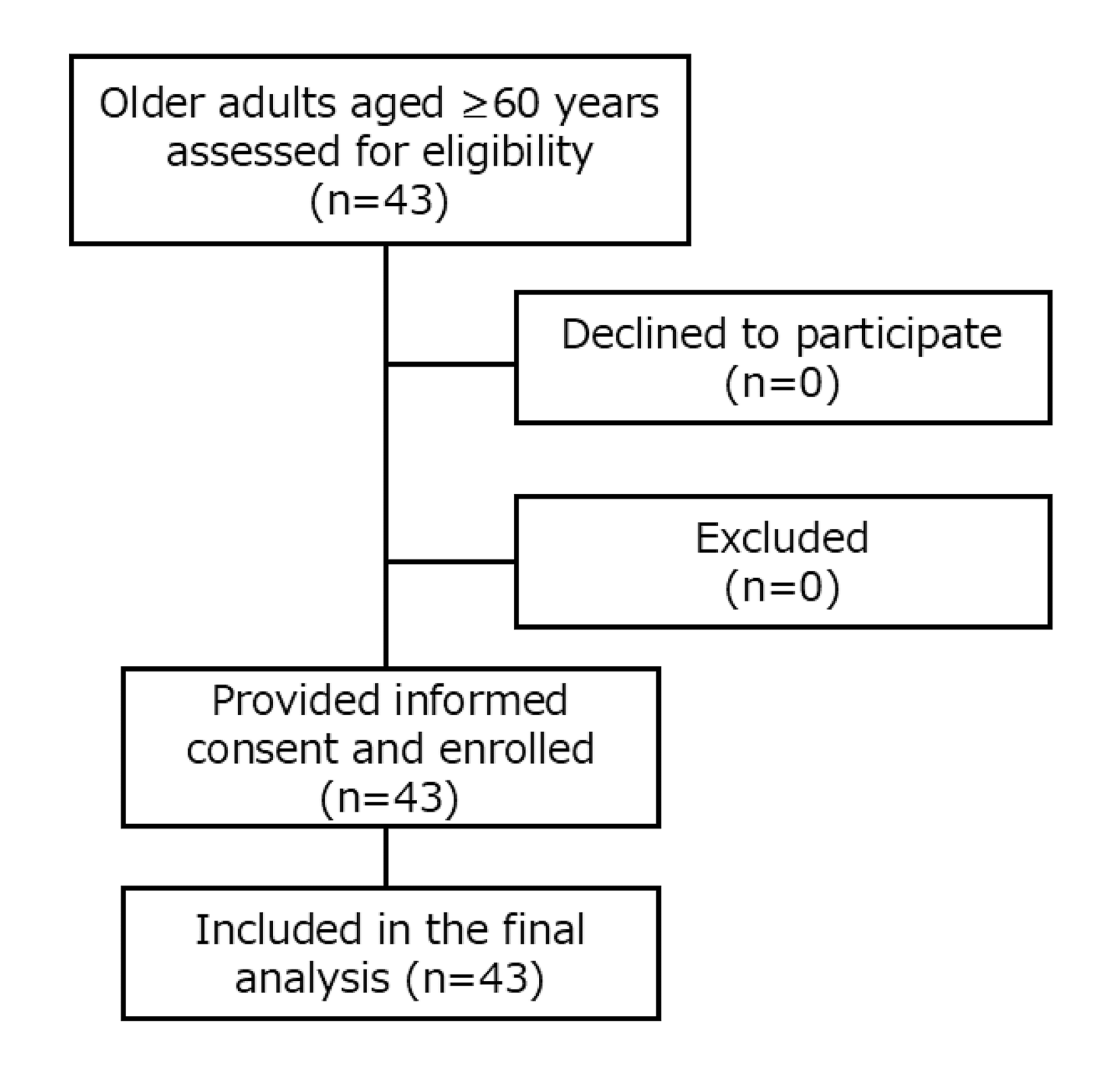
Flow diagram of participant recruitment and inclusion. During the study period, older adults aged ≥60 years who visited the dental clinic were screened for eligibility. After exclusion of individuals who were unable to expectorate saliva or had cognitive impairment preventing tongue pressure measurement, eligible participants were enrolled consecutively. All enrolled participants completed the study procedures, and no dropouts occurred. The final analysis included all enrolled participants.

All eligible participants who were approached agreed to participate in the study, and no participants were excluded or lost during the study period (Figure 1). There were no missing data for any of the variables included in the analysis.

The study population consisted of 16 men and 27 women, with a mean age of 73.7 years. All participants were ambulatory patients who attended a dental clinic for dental treatment; individuals requiring long-term care or those with severe cognitive impairment were not included. The mean tongue pressure was 33.0 kPa, and only three participants had a tongue pressure of <20 kPa. The mean number of remaining teeth was 22.1, and the mean nif-FTU was 7.6, indicating that many participants retained a relatively large number of functional teeth. The other participant characteristics are summarized in Table 1.

**Table 1:** Characteristics of participants. Values are presented as numbers or mean±SD. Salivary flow rate was measured using the Saxon test; Tongue pressure was measured using a tongue pressure measurement device. ODK, oral diadochokinesis; nif-FTU: functional tooth units consisting of natural teeth and implant-supported or fixed prostheses; FE, feeding efficiency; OHI-DI, Oral Hygiene Index-Debris Index

Variable	Value
Age (years)	73.7±6.10
Sex (n)	-
Male	16
Female	27
Tongue pressure (kPa)	33.0±8.88
Tongue coating index	2.00±1.99
OHI-DI	0.85±0.59
Salivary flow rate (Saxon test, g/2 min)	4.29±1.77
ODK rate (times/s)	-
/pa/	5.93±1.29
/ta/	5.97±1.16
/ka/	5.72±1.10
Salivary bacterial count (log10 copies/mL)	5.97±0.54
Number of remaining teeth	22.1±5.20
nif-FTU	7.60±3.62
Bite size (g)	9.94±4.38
Number of chewing strokes per bite	47.3±21.9
FE	24.4±14.8

Relationship between feeding efficiency and oral function measures

**Figure 2:**
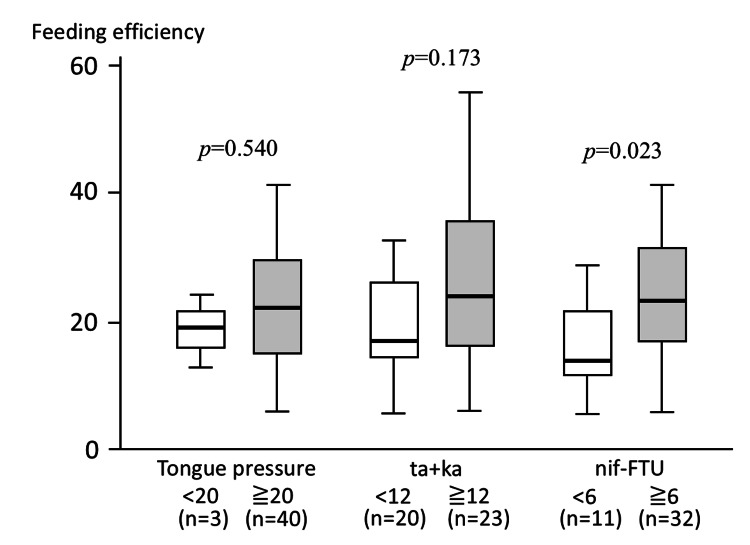
Comparison of FE according to oral function-related parameters. FE values were compared according to tongue pressure (<20 kPa vs. ≥20 kPa), oral ODK (ta+ka <12 vs. ≥12), and nif-FTU (<6 vs. ≥6). Tongue pressure was measured using a tongue pressure measurement device as previously described [11], ODK was assessed using the /ta/ and /ka/ repetition test [12], and nif-FTU was defined based on the number of opposing premolars and molars [13]. Box plots represent the median and interquartile range, with whiskers indicating the minimum and maximum values. FE was significantly higher in participants with nif-FTU ≥6 than in those with nif-FTU <6, whereas no significant differences were observed according to tongue pressure or ODK. FE, feeding efficiency; ODK, oral diadochokinesis; nif-FTU, functional tooth units consisting of natural teeth and implant-supported or fixed prostheses

FE values ranged from 5.6 to 67.7, with a mean±SD of 24.4±14.8. When the relationships between FE and oral function-related parameters (tongue pressure, ODK (ta+ka), and nif-FTU) were examined, FE tended to be lower in participants with a tongue pressure of <20 kPa than in those with ≥20 kPa; however, this difference did not reach statistical significance (*p*=0.540), likely due to the small number of participants with tongue pressure values below 20 kPa, and the results should be interpreted with caution. Participants with ta+ka values ≥12, corresponding to the predefined cutoff value, tended to show higher FE values, although the variability was large and no significant difference was observed (*p*=0.173). In contrast, FE was significantly higher in participants with nif-FTU ≥6 than in those with nif-FTU <6 (*p*=0.023) (Figure 2).

Relationships of feeding efficiency and oral function measures with salivary bacterial counts

**Figure 3:**
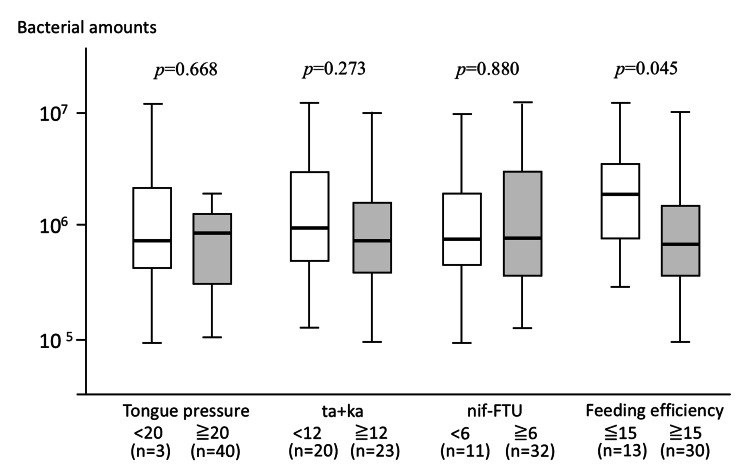
Comparison of salivary bacterial counts according to oral function-related parameters and FE. Salivary bacterial counts were compared according to tongue pressure (<20 kPa vs. ≥20 kPa), ODK (ta+ka <12 vs. ≥12), nif-FTU (<6 vs. ≥6), and FE (<15 vs. ≥15). Tongue pressure was measured using a tongue pressure measurement device as previously described [11], ODK was assessed using the /ta/ and /ka/ repetition test [12], and nif-FTU was defined based on the number of opposing premolars and molars [13]. Box plots represent the median and interquartile range, with whiskers indicating the minimum and maximum values. Salivary bacterial counts were significantly higher in participants with FE <15 than in those with FE ≥15, whereas no significant differences were observed according to tongue pressure, ODK, or nif-FTU. FE, feeding efficiency; ODK, oral diadochokinesis; nif-FTU, functional tooth units consisting of natural teeth and implant-supported or fixed prostheses

Associations between salivary bacterial counts and oral function-related measures were examined. No significant associations were observed between salivary bacterial counts and tongue pressure (*p*=0.668), ta+ka (*p*=0.273), or nif-FTU (*p*=0.880). In contrast, salivary bacterial counts were significantly higher in participants with FE <15 than in those with FE ≥15 (*p*=0.045) (Figure 3).

## Discussion

In this exploratory cross-sectional study of ambulatory older adults, FE varied widely among participants and was significantly associated with the number of FTUs. Lower FE values were associated with higher salivary bacterial counts, whereas conventional single-domain oral function measures, such as tongue pressure and ODK, showed no clear associations with bacterial counts in this relatively healthy population. These findings suggest that FE may capture integrated aspects of the feeding process that are not fully reflected by conventional oral function tests.

The present study suggests that the simple FE test using a wiener sausage reflects overall aspects of oral function during habitual eating and may have potential as a screening indicator for oral functional assessment in older adults. With the progression of population aging, it is well recognized that oral function declines during the transition from robustness to frailty and eventually to a state requiring long-term care. Oral function is an integrated process involving coordinated activities such as mastication, bolus formation and transport by the tongue, and swallowing; impairments in these functions are closely associated with eating and swallowing disorders and an increased risk of aspiration pneumonia.

To date, various methods have been used to evaluate oral function, including tongue pressure measurement, occlusal force measurement, masticatory performance tests, and VE. However, these methods assess specific components of oral function and do not comprehensively capture the entire sequence of feeding behaviors from food intake to swallowing. In addition, many of these examinations require specialized equipment or technical expertise, which may limit their feasibility for routine screening, particularly in older adults with cognitive impairment.

Against this background, several approaches have been reported that assess feeding behavior using actual foods. For example, Zhu et al. [18] measured chewing cycles and chewing time using carrot pieces and electronic recording devices; Wintergerst et al. [19] evaluated the number of chewing cycles required to reach the swallowing threshold using an artificial test food (Cuttersil®); other studies have analyzed the uniformity of color mixing in two-color chewing gum using image analysis [20]; some have assessed particle size and weight after a fixed number of chewing cycles using solid foods such as peanuts or crackers [21,22]; and others have evaluated particle size distribution after a fixed number of chewing cycles using artificial test foods such as Optocal® [23,24]. While these methods are valuable for assessing masticatory performance, many require specialized devices or analytical procedures, limiting their applicability for simple screening in daily clinical practice or community settings.

The FE test developed in this study is characterized by its simplicity, as it uses a commonly consumed food item and requires only counting chewing cycles until swallowing is completed, without the need for special equipment or advanced analysis. Because FE is calculated as the amount of food consumed per chewing cycle, it is expected to decrease when impairments occur in any component of mastication, tongue movement, or swallowing. Thus, FE may serve as an integrated screening indicator reflecting overall FE rather than isolated oral functions.

In this study, FE was significantly higher in participants with a greater number of nif-FTUs, suggesting that the number of functional teeth contributes substantially to FE. In contrast, no clear associations were observed between FE and tongue pressure or ODK. This may be partly attributable to the relatively preserved oral function of the study population, as most participants were ambulatory older adults and only a small number exhibited reduced tongue pressure. Consequently, the sensitivity of FE for detecting severe oral functional decline could not be fully evaluated in the present study.

Notably, salivary bacterial counts were significantly higher in participants with low FE values (<15), whereas no significant associations were observed with individual oral function measures such as tongue pressure or FTUs. This finding suggests that FE may capture complex functional impairments affecting the smooth coordination of the entire feeding and swallowing process, which may be associated with reduced oral self-cleansing and salivary clearance. Although causal relationships cannot be inferred from this cross-sectional study, the observed association indicates that FE may have potential relevance as a screening indicator for aspiration pneumonia risk.

Overall, the FE test appears to be a practical and easily implementable screening method for assessing oral function in routine clinical practice and community settings. Low FE values may indicate the need for further detailed examinations, such as tongue pressure measurement or swallowing function assessments, to identify specific impairments within the feeding and swallowing process and guide appropriate interventions.

Several limitations of this study should be acknowledged. First, the study population consisted of ambulatory older adults with relatively preserved oral function recruited from a single private general dental clinic. Therefore, the findings may not be directly generalizable to older adults receiving institutional care, those with severe frailty, or patients in other healthcare settings. In such populations, FE may be influenced by additional factors not assessed in this study, such as severe sarcopenia, cognitive impairment, swallowing dysfunction, or the need for feeding assistance, which may alter the applicability and interpretation of the FE test. In addition, because this was a cross-sectional study, causal relationships between FE, oral functional decline, salivary bacterial counts, and aspiration pneumonia could not be determined. Second, information on medical comorbidities was not collected, which should be considered when interpreting the results, as systemic health conditions may influence oral function and salivary bacterial counts. Third, the use of a single food item represents a limitation, as differences in food properties may affect FE. In addition, chewing cycles were counted by visual observation, and inter-examiner reliability was not fully assessed. These limitations may have influenced both the direction and magnitude of the observed associations. The inclusion of relatively healthy, ambulatory older adults may have led to an overestimation of FE and its associations with oral function, whereas measurement error related to visual counting of chewing cycles is likely to have introduced non-differential misclassification, which would tend to attenuate the observed associations. Residual confounding due to unmeasured factors, such as medical comorbidities, may have affected the magnitude of the associations, although the direction of this bias cannot be determined. Finally, because this study was exploratory and designed to assess feasibility, the sample size was not determined based on a formal statistical power calculation. Future studies involving frailer populations, multiple food types, standardized measurement protocols, and longitudinal designs are warranted to further validate the clinical applicability of the FE test.

## Conclusions

In this study, we developed a simple FE test using a Wiener sausage and evaluated its potential usefulness as an oral function screening indicator in older adults. FE was associated with the number of FTUs, and low FE values were significantly associated with increased salivary bacterial counts, whereas no such associations were observed with individual oral function indicators such as tongue pressure or ODK.

These findings suggest that FE may reflect overall aspects of the feeding process, encompassing mastication, tongue movement, and swallowing, rather than isolated oral functions. Because the FE test can be easily performed without specialized equipment, it may have potential as a practical screening method for oral function in both clinical and community settings. Further studies involving older adults with more advanced oral functional decline and longitudinal designs are warranted to validate the clinical utility of FE and to explore its potential relevance for assessing aspiration pneumonia risk.
